# The HDAT Helper - Developing an Online Tool to Improve the Safety and Accuracy of Antipsychotic Prescribing

**DOI:** 10.1192/bjo.2022.316

**Published:** 2022-06-20

**Authors:** Christopher Lawrence

**Affiliations:** Southern Health NHS Foundation Trust, Southampton, United Kingdom

## Abstract

**Aims:**

High Dose Antipsychotic Therapy (HDAT) prescriptions and combinations of antipsychotic agents are not currently recommended as standard practice by the RCPsych. College guidance (RCPsych, CR 190) advises that there is “little convincing evidence that off-label prescription of doses of antipsychotic medication above the licensed dosage range has any therapeutic advantage in any clinical setting” and that “any prescription of high-dose antipsychotic medication should be seen as an explicit, time-limited individual trial with a distinct treatment target”. Despite this, both national and local data demonstrate that HDAT has continued to be used regularly across psychiatric inpatient settings, often out of hours and often secondary to the use of PRN Antipsychotics, without a clear treatment plan or rationale. My aim was to create a simple, accessible, online tool that would allow prescribers to quickly and efficiently calculate the BNF percentage of any antipsychotic prescription and that this will enable safer prescribing.

**Methods:**

With the support of a web-developer, I developed an online tool quickly and easily calculate antipsychotic BNF percentages. The tool can be found here: www.hdat.co.uk

**Results:**

The HDAT Helper has been well received at local presentations and I have recently gained the support of senior management at Southern Health NHS Foundation Trust to develop an education programme on HDAT and the HDAT Helper to expand use of the tool across Southern Health.

**Conclusion:**

The current expert guidance, clinical research and my own audit work demonstrates that there are ongoing issues with the prescription of high dose antipsychotics and that at times this occurs inadvertently when different agents are combined.

I believe that the HDAT Helper can make prescribing of antipsychotic agents clearer and more efficient and as a result significantly improve patient safety.

